# Imposter Zoster: An Atypical Case of Varicella-Zoster Virus Infection

**DOI:** 10.7759/cureus.42104

**Published:** 2023-07-18

**Authors:** Galvin Dhaliwal, Waqqas Mirza, John M Vincent Coralde, Jaspreet Dhillon, Taral Patel

**Affiliations:** 1 Internal Medicine, Southwest Healthcare Medical Education Consortium, Temecula, USA; 2 Internal Medicine, Southern California Medical Education Consortium, Temecula, USA; 3 Internal Medicine, Southwest Healthcare Medical Education Consortium, Corona, USA

**Keywords:** antivirals, geriatrics, vaccination, visceral pain, polymerase chain reaction, high-value care, postherpetic neuralgia, abdominal pain, shingles, varicella zoster

## Abstract

In this report, we describe a case involving an 80-year-old female who presented to the emergency department with an acute onset of left upper quadrant abdominal pain. The chief complaint misled us down multiple pathways of considering ischemic bowel disease, peptic ulcer disease, and small bowel obstruction. As a result, this led to costly and invasive diagnostic studies. However, the actual cause eventually became apparent - a cutaneous varicella-zoster virus infection.

This case underscores the significance of maintaining a comprehensive list of potential diagnoses, particularly in elderly adults who commonly present atypically and often face difficulty expressing their symptoms. It also underlines the diagnostic challenges associated with identifying shingles without cutaneous findings. Early detection is crucial in preventing unnecessary tests, minimizing costs, and avoiding treatment delays. Furthermore, the case is a powerful example of the importance of vaccination, which has been proven to be 68-97% effective in preventing shingles and postherpetic neuralgia, depending on the individual's immune function.

## Introduction

Abdominal pain is among the most common presentations in emergency departments, accounting for about 5% of cases [1]. The ambiguity of presentations among the geriatric population makes it more challenging to elicit the cause, as depicted in this case of varicella-zoster virus infection (shingles or herpes zoster). Affecting nearly one million individuals annually, varicella-zoster virus infection results from the reactivation of the latent virus. It remains dormant in the dorsal root ganglia following primary infection with varicella-zoster virus (chickenpox), which usually occurs in childhood. Reactivation can occur at any age but occurs more frequently in the elderly due to age-related decline in cell-mediated immunity [2]. Varicella-zoster virus infection is a painful and disabling disease. In the absence of cutaneous manifestations, diagnosis can be challenging, resulting in increased costs, treatment delays, and diminished quality of life. Here, we present an atypical case of abdominal pain involving varicella-zoster virus infection.

## Case presentation

An 80-year-old female with a history of gastroesophageal reflux disease (GERD), diabetes, hypertension, and asthma presented to the emergency department with non-radiating, postprandial, left upper quadrant (LUQ) and left flank abdominal pain associated with distention, nausea, and constipation that started one week prior to presentation. On arrival, she was afebrile with normal vitals. On the physical exam, no cutaneous findings were noted. She had diminished bowel sounds with tenderness in the LUQ and left flank without rebound tenderness, guarding, or rigidity. Labs were unremarkable except for glucose 149 mg/dL, blood urea nitrogen (BUN) 19 mg/dL, acutely elevated creatinine 1.4 mg/dL, a lactic acid level of 3.0 mmol/L, and a hemoglobin A1c of 9.2%. Urinalysis showed leukocytes and leukocyte esterase. The differential diagnoses at this point included ischemic bowel disease, small bowel obstruction, splenic trauma, peptic ulcer disease, nephrolithiasis, diverticular disease, acute colonic pseudo-obstruction, costochondritis, and severe constipation. A computed tomography (CT) scan with contrast revealed nodular liver and mild colonic diverticulosis with a normal appendix; however, no evidence of bowel obstruction, ischemic bowel disease, or other etiologies mentioned above was found. She reported recently going to urgent care for similar issues and was given omeprazole and sucralfate for possible GERD exacerbation and nitrofurantoin for possible urinary tract infection without relief. 

**Figure 1:**
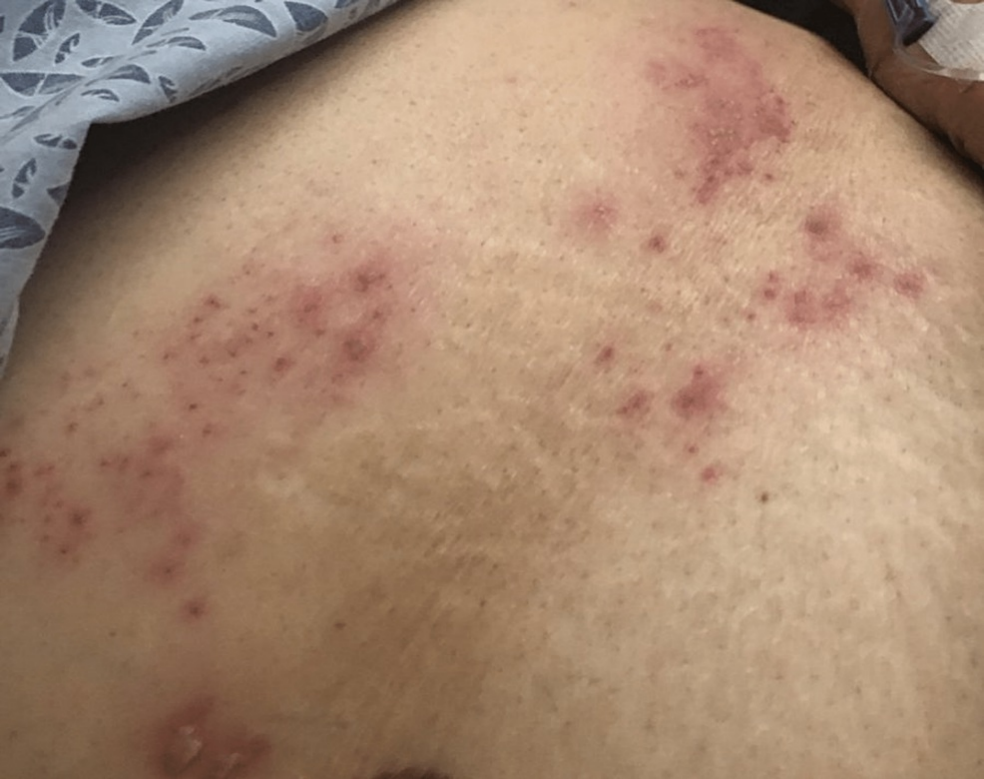
Eruption of vesicular lesions on day five of hospitalization

**Figure 2:**
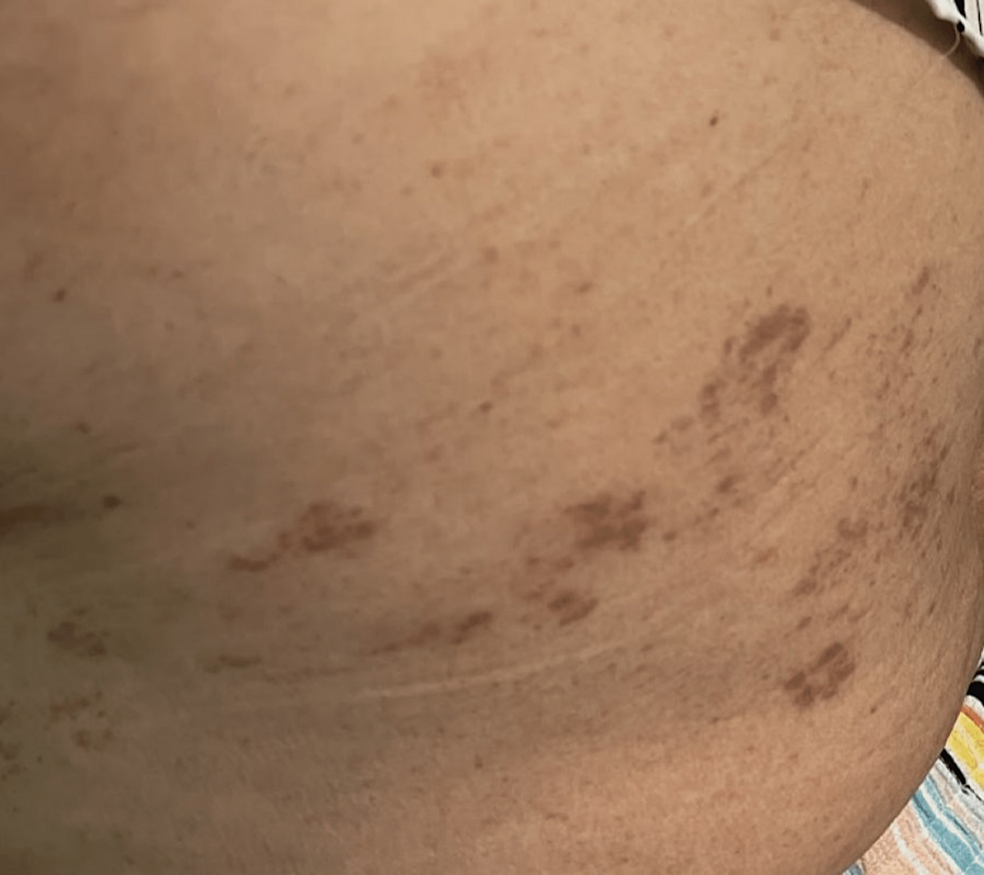
Scarring noted at the site of initial vesicular eruption on two-week follow-up after discharge

She was admitted with supportive management and consultations to both gastroenterology and general surgery. With no surgical intervention required, she underwent an esophagogastroduodenoscopy that did not show any acute findings and was negative for Helicobacter (H.) pylori. Colonoscopy showed non-bleeding internal hemorrhoids, nonbleeding moderate diverticula in the left colon, and a redundant colon, none of which explained the presenting complaints. Her symptoms persisted despite supportive management until day five when vesicular eruptions developed over her LUQ (Figure 1). No skin biopsies were taken because a clinical diagnosis had been obtained from the characteristic vesicular rash in a dermatomal distribution on an erythematous base (Figure 1). She was started on valacyclovir (1000 mg PO every 8 hours for 7 days) and gabapentin (100 mg PO three times a day for 14 days) with moderate symptomatic improvement. On outpatient follow-up two weeks later (Figure 2), she reported persistent abdominal discomfort. However, she denied any other abdominal complaints. She was then treated for postherpetic neuralgia with a prolonged course of gabapentin.

## Discussion

Pathophysiology

Varicella-zoster virus infection is the reactivation of an enveloped double-stranded DNA virus, varicella-zoster, which becomes dormant in the dorsal root ganglia following primary infection during childhood with chickenpox. Reactivation can occur when there is a decline in virus-specific cell-mediated immunity in high-stress states, including increasing age, infections, autoimmune disease, pregnancy, malignancies, and immunomodulatory drugs, among others [3]. The reactivated virus then travels along the sensory nerve, causing a characteristic pattern of pain and skin lesions in a dermatomal distribution. However, multi-dermatomal involvement can be seen in immunocompromised individuals. In cases where the infection progresses to postherpetic neuralgia, the pathophysiology is not fully understood.

Clinical features and diagnosis

Varicella-zoster virus infection can occur at any age; however, incidence and aggressiveness increase with age. The pain caused by herpes zoster is often described with vague presentations such as burning, stabbing, itching, or aching. Misdiagnosis, in the absence of cutaneous findings, can lead to costly consultations with invasive interventions and unfortunate delays in treatment [4]. However, as depicted in our case, the differential should remain high in undifferentiated abdominal pain. For highly suspicious cases, a polymerase chain reaction of blood or stool can be done for early detection [5]. In places where a polymerase chain reaction is unavailable, reports suggest that a CT scan may help identify isolated periarterial fat stranding in visceral varicella-zoster virus infections [6].

Complications can include long-term postherpetic neuralgia, vision loss, bacterial superinfection, and cranial and peripheral nerve palsies [7]. In cases of ocular involvement, ophthalmologic consultation is recommended for proper management due to the potential for severe complications [8]. Postherpetic neuralgia is potentially the most troublesome, with severe itching and allodynia with limited treatment options. These complications can negatively impact the quality of life and strain activities of daily living. In severe cases, the need for hospitalization increases, causing significant costs to the healthcare system.

Treatment

Treating varicella-zoster virus infection and its complications requires a multifaceted approach tailored to the individual's needs. If diagnosed early (within 72 hours of symptom onset), antiviral agents effectively reduce the duration of the rash and help alleviate associated pain. Commonly used agents include acyclovir, valacyclovir, and famciclovir, with valacyclovir showing slightly better results in pain reduction, duration of symptoms, and prevention of postherpetic neuralgia [9]. For analgesia, options include over-the-counter medications, narcotics, neuropathic pain medications, injectable nerve blocks, and topical treatments. With progression to postherpetic neuralgia, additional treatment options include tricyclic antidepressants, anticonvulsants, and nonmedical modalities such as transcutaneous electric nerve stimulation and biofeedback [8].

Prevention

Prevention strategies rely heavily on early vaccinations. In one randomized control trial comprising 38,546 patients by Oxman et al., the use of the zoster vaccine reduced the incidence of herpes zoster by 51.3% (P<0.001), reduced the burden of illness due to herpes zoster by 61.1% (P<0.001), and reduced the incidence of postherpetic neuralgia by 66.5% (P<0.001) [10]. The Centers for Disease Control and Prevention recommends that adults 50 years and older get two doses of the recombinant zoster vaccine [11].

Going back to our patient, we discovered that not only was she immunocompromised due to her age and uncontrolled diabetes, but she was also unvaccinated against the virus despite having a history of chickenpox as a child. We believe this may have contributed to her postherpetic neuralgia. The patient and her family were adequately educated regarding the importance of diabetes management and the necessity of vaccination to prevent such complications.

## Conclusions

This case highlights the need for the early diagnosis and treatment of varicella-zoster virus infection for favorable outcomes and high-value care. Varicella-zoster virus infection should always be considered when investigating patients presenting with acute onset of abdominal pain. In addition, it exemplifies the importance of vaccination, as emphasized above. We recommend and encourage reporting atypical cases of varicella-zoster virus infection to further study unique presentations and improve clinical outcomes.
